# Crizotinib targets in glioblastoma stem cells

**DOI:** 10.1002/cam4.1167

**Published:** 2017-09-27

**Authors:** Audelaure Junca, Claire Villalva, Gaëlle Tachon, Pierre Rivet, Ulrich Cortes, Karline Guilloteau, Anaïs Balbous, Julie Godet, Michel Wager, Lucie Karayan‐Tapon

**Affiliations:** ^1^ Department of Cancer Biology University Hospital of Poitiers Poitiers F‐86021 France; ^2^ Department of Pathology University Hospital of Poitiers Poitiers F‐86021 France; ^3^ INSERM U‐1084, Experimental and Clinical Neurosciences Laboratory, Cellular Therapies in Brain Diseases group University of Poitiers Poitiers F‐86022 France; ^4^ Medicine and Pharmaceutical Science Faculty Poitiers University Poitiers F‐86073 France; ^5^ Department of Neurosurgery University of Poitiers Poitiers F‐86021 France

**Keywords:** ALK, crizotinib, glioblastoma stem cells, glioma stem cell microarray, MET, ROS1

## Abstract

Glioblastoma stem cells (GSCs) are believed to be involved in the mechanisms of tumor resistance, therapeutic failures, and recurrences after conventional glioblastoma therapy. Therefore, elimination of GSCs might be a prerequisite for the development of successful therapeutic strategies. ALK, ROS1, and MET are targeted by Crizotinib, a tyrosine kinase inhibitor which has been approved for treatment of ALK‐rearranged non–small‐cell lung cancer. In this study we investigated ALK, ROS1, and MET status in nine glioblastoma stem cell lines and tumors from which they arise. Fluorescent in situ hybridization (FISH), Sanger's direct sequencing, and immunohistochemistry were used to screen genomic rearrangements (or amplifications), genomic mutations, and protein expression, respectively. The immunohistochemical and FISH studies revealed no significant dysregulation of ROS1 in GSCs and associated tumors. Neither amplification nor polysomy of *ALK* was observed in GSC, but weak overexpression was detected by IHC in three of nine GSCs. Similarly, no *MET* amplification was found by FISH but three GSCs presented significant immunohistochemical staining. No ALK or MET mutation was found by Sanger's direct sequencing. In this study, we show no molecular rearrangement of *ALK, ROS1,* and *MET* that would lead us not to propose, as a valid strategy, the use of crizotinib to eradicate GSCs. However, MET was overexpressed in all GSCs with mesenchymal subtype and three GSCs presented an overexpression of ALK. Therefore, our study corroborates the idea that MET and ALK may assume a role in the tumorigenicity of GSC.

## Introduction

Primary tumors of the central nervous system (CNS) account for about 2–3% of all cancers in humans and gliomas are the most frequent (70%). In the 2007 WHO classification, glioblastomas (GBM), the highest grade of gliomas (Grade IV) are classified among the group of astrocytic tumors. They represent about 49% of brain tumors in adults, almost 30000 new cases per year in Europe 1. There are two types of GBM: primary or de novo glioblastomas, which account for 90% of GBM, and secondary glioblastomas arising on a low‐grade glioma 2, 3. The current standard of care for patients with GBM includes tumor resection followed by adjuvant radiotherapy and chemotherapy. The prognosis is very poor with an average survival of 3 months without treatment, 12.1 months with radiotherapy alone and 14.6 months with Stupp protocol combining radiotherapy, chemotherapy, and temozolomide 4.

According to many studies, GBMs derive from malignant transformation of stem cells and/or glial precursor cells 5, 6, 7. Glioblastoma stem cells (GSC) were identified for the first time in 2002 8. They are rare in tumors (0.5–5% of total tumor cells) and are considered to be responsible for not only the onset and the maintenance of tumors but also their resistance to conventional chemotherapeutic treatments (including temozolomide) and to radiation therapy 9. In vitro, these cells grow as spheres called “neurospheres” and have the ability to self‐renew and to reform themselves after dissociation. They could recapitulate the parental tumor when transplanted into the rodent central nervous system and retain tumor‐forming capacity through serial transplantation 10, 11, 12. It is consequently of particular interest to study new therapeutic targets specifically targeting GSC 13.

Crizotinib is a targeted therapy agent, which has been approved in adults since 2012 for treatment of non–small lung cancer with an activating translocation of *ALK* gene. It was originally developed as an inhibitor of *Mesenchymal‐epithelial transition MET* but is also active against structurally related tyrosine kinases such as *ALK* and *ROS proto‐oncogene 1 ROS1*. It is an orally available ATP‐competitive selective inhibitor that prevents tyrosine phosphorylation on these receptors. Targeted alterations by crizotinib are found in more than 20 different cancers. Since June 2013, as part of a phase 2 clinical trial, the AcSé program of the French National Cancer Institute has been evaluating the efficiency of crizotinib in adult and pediatric cancers presenting an alteration of *ALK, MET,* or *ROS1*. This molecule is proposed to patients with cancer in situations of treatment failure and for whom genetic alterations have been identified in the tumor 14.

The *MET* gene is mapped to the chromosome region 7q31 and encodes a transmembrane tyrosine kinase receptor closely related in sequence to the insulin receptor. It is expressed by cells of epithelial or endothelial origin. The ligand is the hepatocyte growth factor (HGF) expressed by stroma mesenchymal cells and neutrophils. The HGF/MET activation pathway, essential in embryogenesis, plays a role in cell proliferation and cellular migration, particularly in cases of tissue aggression, in order to restore the integrity of injured tissues 15. It is also implicated in tumor development, angiogenesis, and progression to cancer cells with metastatic potential 16. In stem cells, MET is necessary for the transition from the phase G0 to an alert phase that positions stem cells to respond rapidly to any stress condition 17. Different abnormalities in this signaling pathway have been described: overexpression of HGF ligand, overexpression of the receptor, genomic amplification, and misense mutations, especially in exons 14–19. MET is frequently overexpressed in GBM and expression correlates with tumor grade 18. HGF/MET signaling also confers resistance to radiotherapy by promoting survival of glioma stem cells (GSCs) 19. Different MET inhibition strategies are being developed such as HGF ligand or MET receptor inhibitions, particularly with crizotinib, which has shown efficacy in depleting tumor‐propagating stem‐like cells 20.

The *ALK* gene (Anaplastic Lymphoma Kinase) is mapped to the chromosome region 2p23.2 and encodes the ALK protein, a tyrosine kinase receptor (RTK) of the insulin receptor family. *ALK* native transcripts are essentially and transiently expressed during development in specific regions of the central and peripheral nervous systems, such as the thalamus, mid‐brain, olfactory bulb, and peripheral ganglia, and are localized mainly in neuronal cells. As ALK expression is maintained, albeit at a lower level, in the adult brain, it might play an important role in both the normal development and function of the nervous system 21. ALK is expressed at a significantly higher level in high‐grade brain tumors [glioblastoma and anaplastic oligodendrogliomas] when compared to normal brain tissue and low‐grade tumors 22. Decreased growth and increased apoptosis of glioblastoma xenografts in athymic nude mice with ribozyme‐mediated targeting of ALK have been shown to occur 23. Three types of *ALK* alterations have been described in tumors: first, intra, or interchromosomic rearrangements leading to formation of a fusion gene having an oncogene activity—the most common fusion partner being *EML4* (*Echinoderm microtubule‐associated protein‐like 4*)—second, genomic amplifications, and, lastly, mutations especially in exons 20–25. Rearrangements and mutations of *ALK* gene result in ligand‐independent auto‐phosphorylation of the protein and activation of downstream signaling pathways that play a role in cell proliferation and survival. They have been described in anaplastic large‐cell lymphoma, non–small‐cell lung cancer (NSCLC), inflammatory myofibroblastic tumors, diffuse large B‐cell lymphoma, squamous cell carcinoma of the esophagus, and neuroblastoma. *ALK* amplification has been described in neuroblastoma, NSCLC, rhabdomyosarcoma, esophageal cancer, and endometrial carcinosarcomas. Misense mutations are present in neuroblastoma and anaplastic thyroid cancer. Tumors originating from various organs that harbor *ALK* abnormalities have been defined as “*ALK*oma” 24.

The *ROS1* gene is mapped to the chromosome region 6q22.1 and encodes an orphan transmembrane tyrosine kinase receptor phylogenetically related to ALK and the insulin receptor family. It is expressed transiently in various tissues during development with little to no expression in adult tissues. Overexpression has been reported mainly in meningiomas (55%) and glioblastoma multiform (29%). Intra‐ and interchromosomic rearrangements involving *ROS1* have been characterized in cholangiocarcinomas and NSCLC 25. Many fusion partners have been described including FIG (Fused in Glioblastoma also known as Golgi‐associated PDZ and coiled‐coil domain‐containing protein). The 3′ region of *ROS1* gene and the 5′ region of the partner gene generate a gene fusion encoding a chimeric tyrosine kinase protein that initiates an intracellular signal resulting in an oncogenic activation cascade of MAP kinase pathways through phosphorylation of RAS 26. It has been shown that the *ROS1* rearrangement could provide sensitivity to crizotinib and early clinical studies in ROS1‐positive NSCLC patients brought out promising responses 27. To answer the question of whether Crizotinib could be effective in the fight against glioblastoma stem cells, we examined the targetable molecular anomalies in stem cell lines established from patient samples. The amplification and mutations of *MET,* the translocation, amplification, and mutations of *ALK,* and the translocation of *ROS1* have consequently been sought in GSC.

## Materials and Methods

### Cell lines

Nine cell lines of GSC have been investigated. These cell lines were established and characterized in our laboratory as we previously described in detail 28, 29. Briefly, following informed consent, the patients were operated at the University Hospital of Poitiers with de novo glioblastomas diagnosed between 2006 and 2009. The average age of these patients at diagnosis was 62.1 years and average survival was 12.6 months. They had received chemotherapy with temozolomide and, in most cases, radiotherapy (Table 1). Characterization of the tumor was performed by experienced pathologists (Table S1). Tumor sphere cultures were performed and characterized for self‐renewal, differentiation, and in vitro clonogenicity by limiting dilution assays. Tumorigenicity and stemness properties were evaluated by xenograft experiments in nude mice. In this study, cells were used at low passage number 18, 19, 20, 21, 22, 23, 24, 25, 26, 27, 28. Molecular profile, including *MGMT (*O6‐methylguanine‐DNA‐methyltransferase) promoter methylation, *EGFR* copy number, *IDH1*,* IDH2*,* EGFR‐variant III*,* p53*,* PTEN* status, as well as LOH (Loss Of Heterozygosity) at loci 1p36, 19q13, 9p21, and 10q23 was performed and the results are presented in Table S2.

**Table 1:** Patients and corresponding GSCs characteristics

Patients/Cell line	Age	Gender	Overall survival [Months]	Radiotherapy [Gy]	Chemotherapy	WHO classification	Verhaak subtype
1	69	M	14	60	TMZ	Grade IV	Proneural
2	57	M	9	60	TMZ	Grade IV	Neural
3	56	M	9	60	TMZ	Grade IV	Classical
4	66	M	6	Non	TMZ	Grade IV	Proneural
5	65	F	11	60	TMZ	Grade IV	Neural
6	53	M	4	60	TMZ	Grade IV	Mesenchymal
7	69	M	25	40	TMZ	Grade IV	Classical
8	61	M	27	nc	TMZ	Grade IV	Mesenchymal
9	63	M	9	60	TMZ	Grade IV	Mesenchymal

TMZ, Temozolomide; GSCs, glioma stem‐like cell lines.

### Cell microarrays

**Figure 1:**
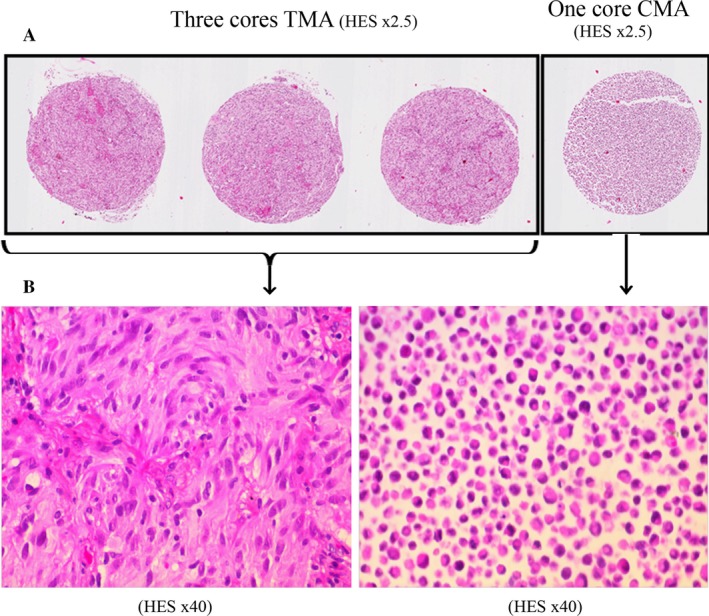
(A) Disposition of three cores of TMA and one core of CMA on one slide (HES coloration × 2,5), (B) Photographs of TMA and CMA cores (HES coloration × 40).

From the nine cell lines, Cell MicroArrays (CMA) were established. They were obtained after fixing stem cells in formalin (10% neutral buffered) and paraffin embedding. A 1‐mm‐diameter biopsy core for each of the nine lines was transferred to the recipient block using a TMA workstation (Alphelys, Plaisir, France) (Fig. 1).

### Tissue microarrays

**Figure 2:**
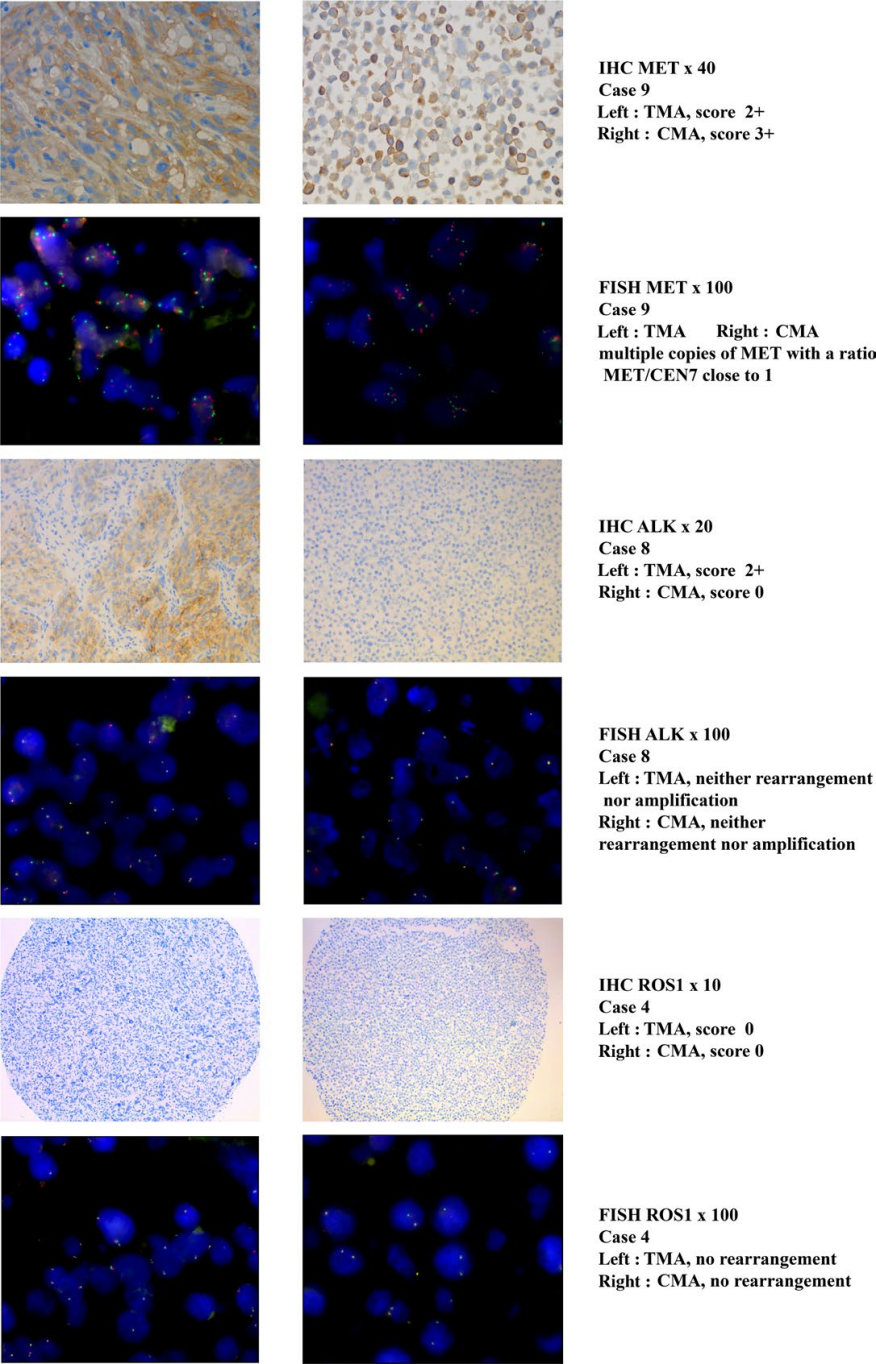
Examples of IHC and FISH results.

Tissue MicroArrays (TMA) were established using paraffin‐embedded tissue samples from tumor biopsies or surgical removal of the same patients. All specimens had >30% tumor content. To overcome tumor heterogeneity, three biopsy cores of 1 mm diameter were included in the same recipient paraffin block as the CMA (Figs. 1, 2).

Slides with a 3‐*μ*m‐thickness section of the TMA/CMA‐embedded paraffin block were produced to achieve immunohistochemistry and Fluorescent in situ hybridization (FISH) experiments for each antibody or probe.

### Immunohistochemistry

ALK immunohistochemistry (IHC) was performed using the prediluted D5F3 monoclonal antibody and the protocol developed by Ventana Rabbit Monoclonal Primary Antibody (ref: 792‐4794). ROS1 immunohistochemistry was performed using the prediluted D4D6 monoclonal antibody and the protocol developed by Genemed Rabbit Monoclonal Primary Antibody (ref: 60‐0180‐7). Cases were assessed as IHC score 0 (no staining), 1+ (faint cytoplasmic staining), 2+ (moderate, smooth cytoplasmic staining), or 3+ (intense, granular cytoplasmic staining) by two expert pathologists. An IHC cytoplasmic staining with an intensity of 1+, 2+, or 3+ on more than 10% of tumor cells was defined as an ALK‐positive result. Any percentage of tumor cells with a cytoplasmic and membrane signal intensity of 1+, 2+, or 3+ was defined as a ROS1‐positive result.

MET immunohistochemistry was performed using the prediluted SP44 monoclonal antibody and the protocol developed by Ventana Rabbit Monoclonal Primary Antibody (ref: 790‐4430). Only strong IHC staining with a 2+ or 3+ intensity present in more than 50% of tumor cells was defined as a MET‐positive result.

### FISH

Analysis of *ALK*,* ROS1,* and *MET* by FISH was conducted using Vysis^®^ LSI ALK Dual Color Break Apart FISH Probe, ZytoLight^®^ ROS1 SPEC Dual Color Break Apart, and ZytoLight^®^ MET/CEN7 Dual Color Probe, respectively. The Zeiss Axio Imager Z2 fluorescent microscope, ×100 objective, and Isis software were used for scoring each case and image acquisition.

Reading was carried out in double filter or alternating red and green filters by two experienced molecular biologists trained in interpretation and applying the interpretative guidelines provided by the manufacturer and the published recommendations 30, 31. A hundred nuclei for each CMA and fifty nuclei for each TMA were counted. FISH locus rearrangements were considered as positive in relation to two different patterns: the classic break‐apart pattern with one fusion signal and two separated orange and green signals (more than two signal diameters apart) and the atypical pattern with a single red (in case of ALK) or green (in case of ROS1) signal in addition to fused signals. FISH‐positive cases for both *ALK* and *ROS1* rearrangements were defined as more than 15% split or single signals. Concerning amplification, an average copy number of *ALK* or *MET* inferior to 6 was considered negative 32.

### DNA extraction

Genomic DNA from cultured cells and from paraffin‐embedded tissue samples was extracted using the DNAeasy Blood and Tissue DNA isolation kit and QIAamp DNA Mini Kit (Qiagen, Hilden, Germany), respectively. The presence of at least 50% tumor cells in paraffin‐embedded tissue samples had previously been evaluated histologically.

### DNA sequencing

Mutations were investigated by bidirectional sequencing using the standard Sanger method. The primer sequences are available on request. We chose to study exons 23, 24, and 25 of *ALK* gene and exons 14, 16, 17, 18, and 19 of *MET* gene because mutations in these exons, coding for the protein kinase domains, have been described as conferring sensitivity or resistance to Crizotinib 33, 34.

## Results

### Study of *MET*

The results of IHC were variable depending on the sample (Table 2, Fig. 2). Two tissue samples (5 and 9) showed positive staining for MET (IHC 3+ and 2+, respectively) but only one (case 9) had a comparable expression in GSC (Table 2). On the contrary, two GSC lines (6 and 8) had a higher expression of MET than in tumor samples. The FISH showed that most tumor samples and GSC lines exhibited polysomy with a mean copy number of 5.3 and 4.2 and a ratio of 1.1 and 1.0, respectively. In particular, we noted that overexpression in case 9 was related to polysomy with a copy number of 7.1 (ratio 1.1) in tumor sample and 5.1 (ratio 1.0) in GSC (Table 2). There was a discrepancy between IHC and FISH results for tumor sample and GSC line 7 with no staining in IHC despite a high copy number of *MET* genes. There was no correlation between copy number in GSC and in associated tumor samples (*P* = 0.4 Spearman). Concerning Sanger's sequencing, no mutation was observed by direct sequencing for exons 14 and 16–19 of the *MET* gene, neither in tumor samples nor in GSC lines.

**Table 2:** Analysis of MET amplification by IHC and FISH on TMA and CMA

			FISH			
MET	IHC		TMA		CMA	
CSG lines	TMA	CMA	Mean gene copy number/nucleus	Ratio	Mean gene copy number/nucleus	Ratio
1	−	−	3,4	1	4,4	1,1
2	−	−	4,4	1	4	1
3	−	−	5	1	4,5	1
4	−	−	4,8	1,1	3,5	1
5	+++	−	5,6	1,1	3,6	1
6	−	++	5,4	1,5	3,9	1,1
7	−	−	6,6	1,4	4,9	1
8	−	++	4,9	0,9	3,8	1
9	++	+++	7,1	1,1	5,1	1
Mean value			5,3	1,1	4,2	1

TMA, tissue microarrays; IHC, immunohistochemistry; CMA, cell microarray; FISH, Fluorescent in situ hybridization.

### Study of *ALK*

The staining obtained by IHC was heterogeneous. An IHC staining 2+ was observed in tissue sample 8 without any staining of the associated GSC line. By contrast, three GSC lines (1, 3, and 9) showed a weak signal 1+ without any staining of the associated tissue samples. In all cases, FISH showed neither rearrangement nor amplification of *ALK* (Table 3). The mean value of *ALK*‐rearranged nuclei and the mean value of copy number of *ALK* gene were identical in tumors and in GSC. No mutation was observed by direct sequencing of the ALK gene for exons 23, 24, and 25 in GSC and tumor samples.

**Table 3:** Analysis of ALK translocation and amplification by IHC and FISH on TMA and CMA

			FISH			
ALK	IHC		TMA		CMA	
GSC lines	TMA	CMA	Percentage of positive nuclei	Mean gene copy number/nucleus	Percentage of positive nuclei	Mean gene copy number/nucleus
1	−	+	2	2,1	0	2,4
2	−	−	2	2,3	6	2,3
3	−	+	0	2,3	0	2,2
4	−	−	0	2,5	0	2
5	−	−	2	2,1	0	2,5
6	−	−	2	2,1	0	2,1
7	−	−	4	2,1	0	2,3
8	++	−	0	2,4	2	2
9	−	+	0	2	4	2,7
Mean value			1,3	2,2	1,3	2,3

TMA, tissue microarrays; IHC, immunohistochemistry; CMA, cell microarray; FISH, Fluorescent in situ hybridization.

### Study of *ROS1*

No staining by IHC was observed in the nine GSC lines or in the tissue samples (Table 4). These results were consistent with FISH, finding no rearrangement profile in *ROS1* gene on any analyzed spots (Fig. 2). No difference in positive nuclei between tumors and GSC was noticed.

**Table 4:** Analysis of ROS1 translocation by IHC and FISH on TMA and CMA

			FISH	
ROS1	IHC		TMA	CMA
GSC lines	TMA	CMA	Percentage of positive nuclei	Percentage of positive nuclei
1	−	−	0	8
2	−	−	0	4
3	−	−	2	0
4	−	−	6	2
5	−	−	4	2
6	−	−	0	4
7	−	−	8	4
8	−	−	6	0
9	−	−	0	2
Mean value			2,9	2,9

TMA, tissue microarrays; IHC, immunohistochemistry; CMA, cell microarray; FISH, Fluorescent in situ hybridization.

## Discussion

The aim of this work was to study the targets of crizotinib: ALK, ROS1, and MET by using IHC, FISH, and direct sequencing in nine GSC lines derived from nine glioblastoma samples. The nine GSC lines were used to establish Cell MicroArray (CMA). Tissue MicroArray (TMA) was established from tumor tissue samples. CMA and TMA are very useful tools to compare samples in the same conditions (especially slice thickness, temperature, dilution of reagents) and they save valuable biological tissue material.

Various studies agree that HGF/MET signaling promotes biological activities, resulting in tumor growth, angiogenesis, and the development of invasive phenotypes, making this receptor an attractive target for potential anticancer treatment 35. The alterations of *MET* gene, including amplification, overexpression, and mutations, have been described in numerous solid tumors and have been associated with a poor prognosis 36, 37.

MET expression has been associated with cells of the stem compartment in several tissues and overexpression of HGF promotes the acquisition of stem‐like properties and malignant progression of glioma tumor cells 38, 39. A recent study showed that EGFR inhibition induces increased MET expression and associated proliferation of GSCs‐expressing pluripotency transcription factors and displaying multilineage potential 40. The classification by gene signature identified by Verhaak et al. indicated that MET‐positive neurospheres belonged mostly to the mesenchymal subtype 2. When neurospheres undergo a differentiative program, MET expression is downregulated and in MET‐positive neurospheres, HGF increases in vitro migration suggesting that MET is implicated in epithelial–mesenchymal transition (EMT), which starts the invasive growth program 41. A study by Jooet al. showed heterogeneous expression of MET in the same tumor with overexpression of MET by the GSC localized along the vessels and areas of necrosis. Inhibition of MET signaling pathway in GSCs disrupted tumor growth and invasiveness both in vitro and in vivo, suggesting that MET activation is required for GSCs 19, 42.

We found overexpression of MET by IHC in two cases of nine tumors, which is comparable to results of Kong *et al*., who showed overexpression in 29% of glioblastomas in a series comprising 62 cases. This overexpression was associated with poor prognosis with a survival of 11.7 months against 14.3 months for patients whose tumors did not express or had low expression of MET 43. The same trend was observed in our series with average survival of 10 and 13.4 months, respectively. For one of the two cases, we found overexpression of MET in GSC and its related tissue. Two other cases showed overexpression only in GSC. Interestingly, these three cases with expression of MET in GSC belong to the mesenchymal subtype, a finding which is coherent with the idea of MET being a mesenchymal marker.

A polysomy was observed for *MET* in all GSC lines and all tissue samples attached to them with a MET/CEP7 ratio close to 1. The average copy number of *MET* was 5.3 copies/nucleus for tissue samples and was lower with 4.2/nucleus for GSC lines. There was no correlation between copy number in GSC and in associated tumor samples (*P* = 0.4). In our series, while increased expression in several cases could be explained by polysomy, it was not verified for all cases. However, the case with the largest copy number in terms of tumors and in GSCs had the highest MET overexpression.

Although overexpression of MET potentially arises from genetic alteration of *MET*, the target potential of *MET* alterations including polysomy, gene amplification, and gene mutation has not been well‐established. The clinical data suggest that *MET* amplification as strictly defined by a *MET/CEP7* ratio of >2.2 has the potential to act as an oncogenic driver and thereby to render at least one subset of affected tumors responsive to MET‐TKIs such as crizotinib 44. The predictive value of low polysomy in relation to the response to MET inhibitors is less certain. Catenacci and colleagues reported a case of complete response in a patient with advanced gastric tumor and MET gene polysomy between four and six copies, but it was associated with a high serum HGF level 45. On the other hand, MET inhibition in patient‐derived xenografts of metastatic colorectal cancer with chromosome 7 low polysomy did not modify tumor growth 46. We also know that breast tumors with an increased copy number for the human epidermal growth factor receptor 2 (HER2) gene as a result of low polysomy 17 behave as HER2‐negative tumors 47.

ALK and its ligand, pleiotrophin, are highly expressed during embryonic brain development and in high‐grade brain tumors (glioblastoma and anaplastic oligodendrogliomas) compared to normal brain tissue and low‐grade tumors and contribute to the growth of glioblastoma 23, 48. In addition, an anti‐ALK antibody has been shown to repress the invasive capacity of glioblastoma cell‐line U87 46. Koyama‐Nasu R et al. have shown that the ALK protein and its ligand pleiotrophin are required for the self‐renewal and tumorigenicity of GSC 49. To our knowledge, the study of the *ALK* rearrangement in GSC lines has never been reported in the literature. No translocations involving ALK have been observed in GSC or in tumors. We have found an expression of ALK in three GSCs and no expression in related tumors, supporting the idea that, consistent with previous reports, ALK plays a role in the maintenance of stemness 50. No association with the Verhaak subtype has been observed. It is worth noting that the tumor with the strongest expression of ALK without expression in GSC corresponded to the patient with the longest overall survival.

*ROS1* rearrangement had previously been observed in the glioblastoma U118MG cell line by Charest A et al.*,* who first found a microdeletion on 6q21 region responsible for the fusion of *FIG,* a gene coding for a Golgi apparatus‐associated protein to the kinase domain of the proto‐oncogene ROS1. The fused protein product FIG‐ROS leads to constitutive kinase activation and results in oncogenic transformation 51. A study conducted in our laboratory was designed to determine the prevalence of *FIG‐ROS1* rearrangement in 268 cases of gliomas. Our data suggested that this particular fusion is not present or has a relatively low occurrence in both high‐grade gliomas (<0,6%) and low‐grade gliomas (<1%) 52. In our study, we analyzed by FISH not only rearrangement *FIG‐ROS1* but all rearrangements involving *ROS1*. No tumors or GSCs were positive. Concerning the expression of ROS1 in gliomas, the results were contradictory. ROS1 was shown to be overexpressed in 30% of gliomas and dependent upon the methylation of its promoter 53. Our study showed no expression of ROS1 in the nine tumors or in the associated GSC and, according to our knowledge, study of the ROS1 expression on GSC lines has never been reported in the literature.

Cancer stem cells identified and isolated from solid tumors are interesting prospects in terms of targeted therapies, especially in glioblastoma; their involvement in the recurrence of the disease does not appear to require any additional demonstration. Even if no molecular rearrangement of *ALK*,* ROS1,* and *MET* was found in our study, several GSCs presented MET or ALK overexpression, which corroborates the important role MET and ALK may play in tumorigenicity of GSC.

## Conflict of Interest

None declared.

## Supporting information


**Table S1.** Initial pathological and molecular characterization of patients' original glioblastoma.
**Table S2.** Molecular characteristics of glioblastoma stem cells.

## References

[1] Visser, O. , E. Ardanaz , L. Botta , M. Sant , A. Tavilla , P. Minicozzi , et al. 2015 Survival of adults with primary malignant brain tumours in Europe; Results of the EUROCARE‐5 study. Eur. J. Cancer 51:2231–41. 26421825 10.1016/j.ejca.2015.07.032

[2] Verhaak, R. G. , K. A. Hoadley , E. Purdom , V. Wang , Y. Qi , M. D. Wilkerson , et al. 2010 Integrated genomic analysis identifies clinically relevant subtypes of glioblastoma characterized by abnormalities in PDGFRA, IDH1, EGFR, and NF1. Cancer Cell 17:98–110. 20129251 10.1016/j.ccr.2009.12.020 PMC2818769

[3] Figarella‐Branger, D. , C. Colin , A. Tchoghandjian , and N. Baeza . 2010 Bouvier C [Glioblastomas: gliomagenesis, genetics, angiogenesis, and microenvironment]. Neurochirurgie 56:441–448. 20817192 10.1016/j.neuchi.2010.07.010

[4] Stupp, R. , W. P. Mason , M. J. van den Bent , M. Weller , B. Fisher , M. J. Taphoorn , et al. 2005 Radiotherapy plus concomitant and adjuvant temozolomide for glioblastoma. N. Engl. J. Med. 352:987–996. 15758009 10.1056/NEJMoa043330

[5] Hemmati, H. D. , I. Nakano , J. A. Lazareff , M. Masterman‐Smith , D. H. Geschwind , M. Bronner‐Fraser , et al. 2003 Cancerous stem cells can arise from pediatric brain tumors. Proc. Natl Acad. Sci. USA 100:15178–15183. 14645703 10.1073/pnas.2036535100 PMC299944

[6] Singh, S. K. , C. Hawkins , I. D. Clarke , J. A. Squire , J. Bayani , T. Hide , et al. 2004 Identification of human brain tumour initiating cells. Nature 432:396–401. 15549107 10.1038/nature03128

[7] Hadjipanayis, C. G. , and E. G. Van Meir . 2009 Brain cancer propagating cells: biology, genetics and targeted therapies. Trends Mol. Med. 15:519–530. 19889578 10.1016/j.molmed.2009.09.003 PMC2782740

[8] Ignatova, T. N. , V. G. Kukekov , E. D. Laywell , O. N. Suslov , F. D. Vrionis , and D. A. Steindler . 2002 Human cortical glial tumors contain neural stem‐like cells expressing astroglial and neuronal markers in vitro. Glia 39:193–206. 12203386 10.1002/glia.10094

[9] Kang, M. K. , and S. K. Kang . 2007 Tumorigenesis of chemotherapeutic drug‐resistant cancer stem‐like cells in brain glioma. Stem Cells Dev. 16:837–847. 17999604 10.1089/scd.2007.0006

[10] Laks, D. R. , M. Masterman‐Smith , K. Visnyei , B. Angenieux , N. M. Orozco , I. Foran , et al. 2009 Neurosphere formation is an independent predictor of clinical outcome in malignant glioma. Stem Cells 27:980–987. 19353526 10.1002/stem.15 PMC3177534

[11] Galli, R. , E. Binda , U. Orfanelli , B. Cipelletti , A. Gritti , S. De Vitis , et al. 2004 Isolation and characterization of tumorigenic, stem‐like neural precursors from human glioblastoma. Can. Res. 64:7011–7021. 10.1158/0008-5472.CAN-04-1364 15466194

[12] Lee, J. , S. Kotliarova , Y. Kotliarov , A. Li , Q. Su , N. M. Donin , et al. 2006 Tumor stem cells derived from glioblastomas cultured in bFGF and EGF more closely mirror the phenotype and genotype of primary tumors than do serum‐cultured cell lines. Cancer Cell 9:391–403. 16697959 10.1016/j.ccr.2006.03.030

[13] Liu, G. , X. Yuan , Z. Zeng , P. Tunici , H. Ng , I. R. Abdulkadir , et al. 2006 Analysis of gene expression and chemoresistance of CD133 + cancer stem cells in glioblastoma. Mol. Cancer. 5:67. 17140455 10.1186/1476-4598-5-67 PMC1697823

[14] Vassal, G. , and G. Schleiermacher . 2014 Actualités pharmacologiques – le crizotinib (Xalkori^®^). Revue d'oncologie hématologie pédiatrique 2:46–53.

[15] Ruppert, A. M. , M. Beau‐Faller , L. Belmont , A. Lavole , V. Gounant , J. Cadranel , et al. 2011 A simple view on lung cancer biology: the MET pathway. Rev. Mal. Respir. 28:1241–1249. 22152933 10.1016/j.rmr.2011.05.014

[16] Birchmeier, C. , W. Birchmeier , E. Gherardi , and G. F. Vande Woude . 2003 Met, metastasis, motility and more. Nat. Rev. Mol. Cell Biol. 4:915–925. 14685170 10.1038/nrm1261

[17] Rodgers, J. T. , K. Y. King , J. O. Brett , M. J. Cromie , G. W. Charville , K. K. Maguire , et al. 2014 mTORC1 controls the adaptive transition of quiescent stem cells from G0 to G(Alert). Nature 510:393–396. 24870234 10.1038/nature13255 PMC4065227

[18] Koochekpour, S. , M. Jeffers , S. Rulong , G. Taylor , E. Klineberg , E. A. Hudson , et al. 1997 Met and hepatocyte growth factor/scatter factor expression in human gliomas. Can. Res. 57:5391–5398. 9393765

[19] Joo, K. M. , J. Jin , E. Kim , K. Ho Kim , Y. Kim , B. Gu Kang , et al. 2012 MET signaling regulates glioblastoma stem cells. Can. Res. 72:3828–3838. 10.1158/0008-5472.CAN-11-3760 22617325

[20] Rath, P. , B. Lal , O. Ajala , Y. Li , S. Xia , J. Kim , et al. 2013 In Vivo c‐Met Pathway Inhibition Depletes Human Glioma Xenografts of Tumor‐Propagating Stem‐Like Cells. Transl. Oncol. 6:104–111. 23556031 10.1593/tlo.13127 PMC3612837

[21] Morris, S. W. , C. Naeve , P. Mathew , P. L. James , M. N. Kirstein , X. Cui , et al. 1997 ALK, the chromosome 2 gene locus altered by the t(2;5) in non‐Hodgkin's lymphoma, encodes a novel neural receptor tyrosine kinase that is highly related to leukocyte tyrosine kinase (LTK). Oncogene 14:2175–2188. 9174053 10.1038/sj.onc.1201062

[22] Grzelinski, M. , F. Steinberg , T. Martens , F. Czubayko , K. Lamszus , and A. Aigner . 2009 Enhanced antitumorigenic effects in glioblastoma on double targeting of pleiotrophin and its receptor ALK. Neoplasia 11:145–156. 19177199 10.1593/neo.81040 PMC2631139

[23] Stylianou, D. C. , A. Auf der Maur , D. P. Kodack , R. T. Henke , S. Hohn , J. A. Toretsky , et al. 2009 Effect of single‐chain antibody targeting of the ligand‐binding domain in the anaplastic lymphoma kinase receptor. Oncogene 28:3296–3306. 19633684 10.1038/onc.2009.184 PMC4312131

[24] Mano, H. 2012 ALKoma: a cancer subtype with a shared target. Cancer Discov. 2:495–502. 22614325 10.1158/2159-8290.CD-12-0009

[25] El‐Deeb, I. M. , K. H. Yoo , and S. H. Lee . 2011 ROS receptor tyrosine kinase: a new potential target for anticancer drugs. Med. Res. Rev. 31:794–818. 20687158 10.1002/med.20206

[26] Chin, L. P. , R. A. Soo , R. Soong , and S. H. Ou . 2012 Targeting ROS1 with anaplastic lymphoma kinase inhibitors: a promising therapeutic strategy for a newly defined molecular subset of non‐small‐cell lung cancer. J. Thorac. Oncol. 7:1625–1630. 23070242 10.1097/JTO.0b013e31826baf83

[27] Davies, M. 2014 New modalities of cancer treatment for NSCLC: focus on immunotherapy. Cancer Manag. Res. 6:63–75. 24520205 10.2147/CMAR.S57550 PMC3917949

[28] Villalva, C. , S. Martin‐Lanneree , U. Cortes , F. Dkhissi , M. Wager , A. Le Corf , et al. 2011 STAT3 is essential for the maintenance of neurosphere‐initiating tumor cells in patients with glioblastomas: a potential for targeted therapy? Int. J. Cancer 128:826–838. 20473906 10.1002/ijc.25416

[29] Balbous, A. , U. Cortes , K. Guilloteau , C. Villalva , S. Flamant , A. Gaillard , et al. 2014 A mesenchymal glioma stem cell profile is related to clinical outcome. Oncogenesis 3:e91. 24637491 10.1038/oncsis.2014.5 PMC4038390

[30] Thunnissen, E. , L. Bubendorf , M. Dietel , G. Elmberger , K. Kerr , F. Lopez‐Rios , et al. 2012 EML4‐ALK testing in non‐small cell carcinomas of the lung: a review with recommendations. Virchows Arch. 461:245–257. 22825000 10.1007/s00428-012-1281-4 PMC3432214

[31] Bubendorf, L. , R. Buttner , F. Al‐Dayel , M. Dietel , G. Elmberger , K. Kerr , et al. 2016 Testing for ROS1 in non‐small cell lung cancer: a review with recommendations. Virchows Arch. 469:489–503. 27535289 10.1007/s00428-016-2000-3 PMC5082594

[32] Zito Marino, F. , G. Rocco , A. Morabito , C. Mignogna , M. Intartaglia , G. Liguori , et al. 2016 A new look at the ALK gene in cancer: copy number gain and amplification. Expert Rev. Anticancer Ther. 16:493–502. 26943457 10.1586/14737140.2016.1162098

[33] Roskoski, R. Jr . 2013 Anaplastic lymphoma kinase (ALK): structure, oncogenic activation, and pharmacological inhibition. Pharmacol. Res. 68:68–94. 23201355 10.1016/j.phrs.2012.11.007

[34] Cui, J. J. 2014 Targeting receptor tyrosine kinase MET in cancer: small molecule inhibitors and clinical progress. J. Med. Chem. 57:4427–4453. 24320965 10.1021/jm401427c

[35] Ma, P. C. , G. Maulik , J. Christensen , and R. Salgia . 2003 c‐Met: structure, functions and potential for therapeutic inhibition. Cancer Metastasis Rev. 22:309–325. 12884908 10.1023/a:1023768811842

[36] Bonine‐Summers, A. R. , M. E. Aakre , K. A. Brown , C. L. Arteaga , J. A. Pietenpol , H. L. Moses , et al. 2007 Epidermal growth factor receptor plays a significant role in hepatocyte growth factor mediated biological responses in mammary epithelial cells. Cancer Biol. Ther. 6:561–570. 17495520 10.4161/cbt.6.4.3851 PMC3395216

[37] Miller, C. T. , L. Lin , A. M. Casper , J. Lim , D. G. Thomas , M. B. Orringer , et al. 2006 Genomic amplification of MET with boundaries within fragile site FRA7G and upregulation of MET pathways in esophageal adenocarcinoma. Oncogene 25:409–418. 16186806 10.1038/sj.onc.1209057

[38] Boccaccio, C. , and P. M. Comoglio . 2006 Invasive growth: a MET‐driven genetic programme for cancer and stem cells. Nat. Rev. Cancer 6:637–645. 16862193 10.1038/nrc1912

[39] Li, Y. , A. Li , M. Glas , B. Lal , M. Ying , Y. Sang , et al. 2011 c‐Met signaling induces a reprogramming network and supports the glioblastoma stem‐like phenotype. Proc. Natl Acad. Sci. USA 108:9951–9956. 21628563 10.1073/pnas.1016912108 PMC3116406

[40] Jun, H. J. , R. T. Bronson , and A. Charest . 2014 Inhibition of EGFR induces a c‐MET‐driven stem cell population in glioblastoma. Stem Cells 32:338–348. 24115218 10.1002/stem.1554 PMC4442493

[41] De Bacco, F. , E. Casanova , E. Medico , S. Pellegatta , F. Orzan , R. Albano , et al. 2012 The MET oncogene is a functional marker of a glioblastoma stem cell subtype. Can. Res. 72:4537–4550. 10.1158/0008-5472.CAN-11-3490 22738909

[42] Tasaki, T. , M. Fujita , T. Okuda , A. Yoneshige , S. Nakata , K. Yamashita , et al. 2016 MET Expressed in Glioma Stem Cells Is a Potent Therapeutic Target for Glioblastoma Multiforme. Anticancer Res. 36:3571–3577. 27354625

[43] Kong, D. S. , S. Y. Song , D. H. Kim , K. M. Joo , J. S. Yoo , J. S. Koh , et al. 2009 Prognostic significance of c‐Met expression in glioblastomas. Cancer 115:140–148. 18973197 10.1002/cncr.23972

[44] Kawakami, H. , I. Okamoto , W. Okamoto , J. Tanizaki , K. Nakagawa , and K. Nishio . 2014 Targeting MET Amplification as a New Oncogenic Driver. Cancers 6:1540–1552. 25055117 10.3390/cancers6031540 PMC4190554

[45] Catenacci, D. V. , L. Henderson , S. Y. Xiao , P. Patel , R. L. Yauch , P. Hegde , et al. 2011 Durable complete response of metastatic gastric cancer with anti‐Met therapy followed by resistance at recurrence. Cancer Discov. 1:573–579. 22389872 10.1158/2159-8290.CD-11-0175 PMC3289149

[46] Galimi, F. , D. Torti , F. Sassi , C. Isella , D. Cora , S. Gastaldi , et al. 2011 Genetic and expression analysis of MET, MACC1, and HGF in metastatic colorectal cancer: response to met inhibition in patient xenografts and pathologic correlations. Clin. Cancer Res. 17:3146–3156. 21447729 10.1158/1078-0432.CCR-10-3377

[47] Vanden Bempt, I. , P. Van Loo , M. Drijkoningen , P. Neven , A. Smeets , M. R. Christiaens , et al. 2008 Polysomy 17 in breast cancer: clinicopathologic significance and impact on HER‐2 testing. J. Clin. Oncol. 26:4869–4874. 18794552 10.1200/JCO.2007.13.4296

[48] Powers, C. , A. Aigner , G. E. Stoica , K. McDonnell , and A. Wellstein . 2002 Pleiotrophin signaling through anaplastic lymphoma kinase is rate‐limiting for glioblastoma growth. J. Biol. Chem. 277:14153–14158. 11809760 10.1074/jbc.M112354200

[49] Koyama‐Nasu, R. , R. Haruta , Y. Nasu‐Nishimura , K. Taniue , Y. Katou , K. Shirahige , et al. 2014 The pleiotrophin‐ALK axis is required for tumorigenicity of glioblastoma stem cells. Oncogene 33:2236–2244. 23686309 10.1038/onc.2013.168

[50] Dirks, W. G. , S. Fahnrich , Y. Lis , E. Becker , R. A. MacLeod , and H. G. Drexler . 2002 Expression and functional analysis of the anaplastic lymphoma kinase (ALK) gene in tumor cell lines. Int. J. Cancer 100:49–56. 12115586 10.1002/ijc.10435

[51] Charest, A. , K. Lane , K. McMahon , J. Park , E. Preisinger , H. Conroy , et al. 2003 Fusion of FIG to the receptor tyrosine kinase ROS in a glioblastoma with an interstitial del(6)(q21q21). Genes Chromosom. Cancer 37:58–71. 12661006 10.1002/gcc.10207

[52] Karayan‐Tapon, L. , U. Cortes , P. Rivet , C. Jermidi , G. Vassal , M. Wager , et al. 2014 Lack of GOPC‐ROS1 (FIG‐ROS1) rearrangement in adult human gliomas. Eur. J. Cancer 50:2364–2366. 24999209 10.1016/j.ejca.2014.06.001

[53] Jun, H. J. , S. Woolfenden , S. Coven , K. Lane , R. Bronson , D. Housman , et al. 2009 Epigenetic regulation of c‐ROS receptor tyrosine kinase expression in malignant gliomas. Can. Res. 69:2180–2184. 10.1158/0008-5472.CAN-08-3351 19276365

