# Supplementary material for: Crizotinib targets in glioblastoma stem cells
Source: Cancer Med. 2017 Sep 27;6(11):2625–34. doi: 10.1002/cam4.1167 (PMC5673924; doi:10.1002/cam4.1167)
Supplement: Supplementary file 1 — Table S1. Initial pathological and molecular characterization of patients' original glioblastoma. Table S2. Molecular characteristics of glioblastoma stem cells. [file CAM4-6-2625-s001.docx]

**APPENDIX**

**Table S1. Initial pathological and molecular characterization of patients’ original glioblastoma.** LOH, Loss Of Heterozygosity; IDH, Isocitrate Dehydrogenase; WT, Wildtype; GFAP, Glial fibrillary acidic protein; NA: Information Not Available

| **GBM** | **Initial  pathologist diagnosis** | **GFAP** | **Synaptophysin** | **Neurofilament** | **Ki67 (% of cells)** | **Olig2** | **IDH1/IDH2** | **MGMT** | **LOH 1p** | | **LOH 19q** | |
| --- | --- | --- | --- | --- | --- | --- | --- | --- | --- | --- | --- | --- |
| 1 | GBM | positive | negative | negative | 30% | NA | WT | 1,6% | no | no | |  |
| 2 | GBM | NA | NA | NA | NA | NA | WT | 2% | no | no | |  |
| 3 | GBM | positive | positive | positive | 35% | NA | WT | 3,6% | no | yes | |  |
| 4 | GBM | negative | NA | NA | 30% | NA | WT | 23,8% | no | no | |  |
| 5 | GBM | NA | NA | NA | NA | NA | WT | 2% | no | no | |  |
| 6 | GBM | NA | NA | NA | NA | NA | WT | 11% | no | no | |  |
| 7 | GBM | positive | negative | negative | >20% | positive | WT | 39,8% | no | yes | |  |
| 8 | GBM | positive | NA | negative | 20% | positive | WT | 25,4% | no | no | |  |
| 9 | GBM | positive | NA | NA | 20% | positive | WT | 1,8% | no | no | |  |

**Table S2. Molecular characteristics of Glioblastoma Stem Cells** LOH, Loss Of Heterozygosity; IDH, Isocitrate Dehydrogenase; WT, Wildtype; Mut, Mutant; PTEN, Phosphatase and TENsin homolog; EGFR, Epidermal Growth Factor Receptor; MGMT, O-6-methylguanine-DNA methyltransferase; IQ, insufficient quantity.

| **GSC** | **IDH1**  **/IDH2** | **LOH 1p** | **LOH 19q** | **LOH 9p21** | **LOH 10q** | **p53**  **mutation** | **EGFR VIII** | **EGFR  amplification** | **PTEN fragment 1** | **PTEN fragment 2** | **PTEN fragment 3** | **PTEN fragment 4** | **MGMT** |
| --- | --- | --- | --- | --- | --- | --- | --- | --- | --- | --- | --- | --- | --- |
| 1 | WT | no | no | no | yes | no | yes | polysomy | WT | p.P30L p.Y68H   p.F81S p.C83Y | p.L182V   p.L186V p.I224M  p.R234W | p.F341V | 4,4% |
| 2 | WT | no | no | yes | yes | no | no | polysomy | WT | p.A39T | WT | WT | 1,6% |
| 3 | WT | no | yes | yes | yes | no | yes | trisomy 7  + amplification | WT | WT | 225Y>I | WT | 0,07 |
| 4 | WT | no | no | yes | yes | p.C176Y | yes | duplication | WT | c.217_220del4 | WT | WT | 51,2% |
| 5 | WT | no | no | yes | yes | p.R273C p.R335H p.R282W | yes | duplication | WT | WT | WT | WT | 0,02 |
| 6 | WT | no | no | yes | yes | p.V156F | yes | trisomy 7 | WT | p. E40fs | WT | WT | 34,2% |
| 7 | WT | no | yes | yes | yes | no | yes | trisomy 7 | WT | WT | WT | p.R335fs | 86,8% |
| 8 | WT | no | no | IQ | IQ | no | yes | polysomy | WT | WT | WT | WT | 94,2% |
| 9 | WT | no | no | yes | yes | no | yes | trisomy 7 | WT | WT | WT | WT | 18,8% |
